# Neural Network Assisted Estimation for the Structural Nested Accelerated Failure Time Models

**DOI:** 10.1002/sim.70467

**Published:** 2026-04-02

**Authors:** Yiming Chen, Tianzhou Ma, Paul Smith, Takumi Saegusa

**Affiliations:** ^1^ Department of Epidemiology and Biostatistics University of Maryland College Park Maryland USA; ^2^ Department of Mathematics University of Maryland College Park Maryland USA

**Keywords:** causal inference, g‐estimation, recurrent neural network, structural nested accelerated failure time model, time‐to‐event, time‐varying confounding

## Abstract

Time‐varying confounding complicates the causal survival analysis for longitudinal data. Traditional survival models that adjust for time‐dependent covariates fail to estimate the intervention causal effect unbiasedly. The Structural Nested Accelerated Failure Time Model (SNAFTM) can address this challenge effectively. This model estimates the intervention causal effect as the acceleration factor of the survival time while controlling for the time‐varying confounders. However, the SNAFTM model usually relies on the G‐estimation, which lacks power and suffers from computational burden, especially when the model input data is high‐dimensional with a temporally connected nature. This manuscript presents two Neural Networks based algorithms (GE‐SCORE and GE‐MIMIC) that estimate the SNAFTM. These two algorithms can handle high‐dimensional input data while providing less biased and individualized intervention causal effect estimation, as demonstrated by simulations. The proposed algorithms were also applied to a real observational dataset (CARDIA), and we successfully identified and quantified subjects' smoking causal effects on the time to first cardiovascular events.

**Abbreviations**
AFT: accelerated failure time
BP: blood pressure
CARDIA: coronary artery risk development in young adults (study)
CIT: conditional independence testing
CVD: cardiovascular disease
DAG: directed acyclic graph
IPCW: inverse probability of censoring weights
LSTM: long short‐term memory
ML: machine learning
MSM: marginal structural model
NN: neural network
RNN: recurrent neural network
SNM: structural nested model
SNAFTM: structural nested accelerated failure time model
SRA: sequential randomization assumption

## 1 Introduction

**FIGURE 1:**
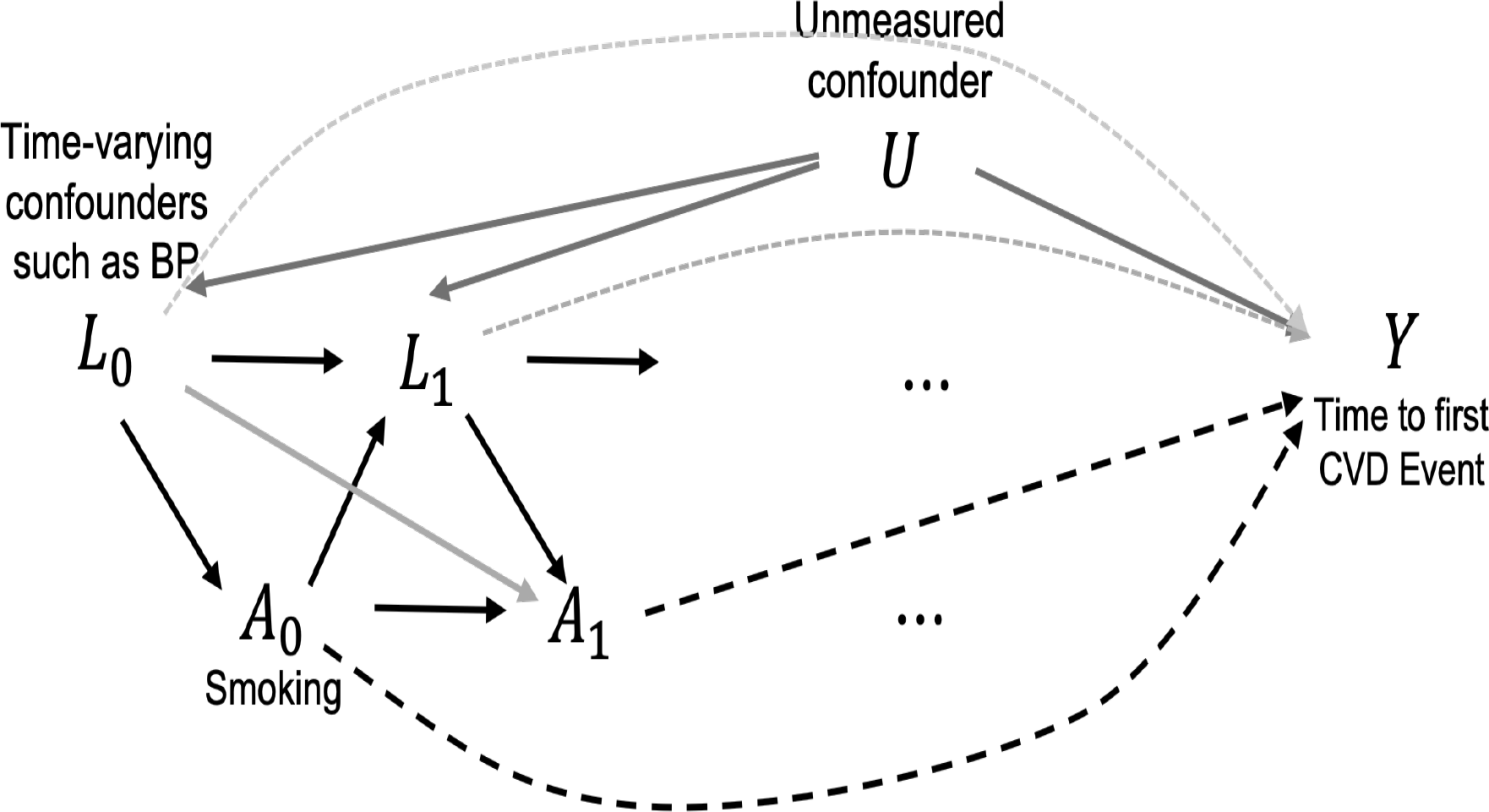
An illustrative DAG for the motivating example.

In longitudinal observational studies, it is common that the exposure or intervention under investigation influences a time‐varying covariate at a later time point, yet this covariate influences the exposure or intervention at subsequent time points. This is known as the time‐varying confounding issue [1]. Considering a crucial research problem of quantifying the causal effect of smoking on the time to subjects' first cardiovascular disease (CVD) event, the existence of time‐varying confounders, such as Blood Pressure (BP), poses a special challenge in choosing the appropriate analysis model. Figure 1 provides a simplified Directed Acyclic Graph (DAG) for this motivating example. Specifically, BP at a certain time t is not only a direct risk factor for individuals' future CVD events, but it is also influenced by individuals' previous smoking status. Furthermore, the historical value of this covariate is also a predictor for future exposure. For instance, a subject may quit smoking after a BP abnormality comes to awareness. As a result, the causal effect of smoking (A) on the time to the first CVD event Y can only be identified when all time‐varying confounders $L_{k}$ (e.g., BP) are controlled appropriately.

The Structural Nested Model (SNM) [2] is an effective method that addresses the time‐varying confounding issue. The SNM models the degree to which the effect of current treatment is modified by past treatment and covariate history. Conceptually, in a longitudinal study with K visits, the active subjects at each visit are a subset of those at the previous visit. Thus, one can construct K+1 sub‐models for such a study, one for each visit. The analysis population of each model is nested within the population of the model at an earlier time point. This is where the “Nested” comes from. When the outcome of interest is a time‐to‐event variable, the Structural Nested Accelerated Failure Time Model (SNAFTM) [3, 4]—an important subclass of SNM, is often used. The SNAFTM estimates the causal effect of the time‐dependent exposure variables on the time‐to‐event outcome, with appropriate control for any time‐varying confounders. It has been successfully applied in numerous clinical settings such as lung transplant and obesity [5, 6]. This model offers some unique benefits in contrast to standard methods, for example, the Cox proportional hazards model. First, hazard has been criticized for decades that it lacks causal meaning. Second, the Cox model would yield biased estimates of the joint exposure effect on the hazard of survival, whether or not it adjusts for the aforementioned time‐varying variables [4].

Yet, the application of the SNM is seriously limited in modern health studies, facing challenges such as the curse of dimensionality and computational complexity. Magnitudes of data (especially for the risk factors L) collected are growing rapidly in modern health studies. Consequently, as a benchmark method, G‐estimation for structural nested models is susceptible to model misspecification and imposes considerable computational demands. Furthermore, G‐estimation usually obtains a population‐level intervention causal effect, which ignores heterogeneity. Machine learning algorithms are known for their ability to capture complex, non‐linear relationships in high‐dimensional data, thus leading to more accurate and flexible predictions. There are successful applications of machine learning algorithms to AFT model estimation [7]. Also, neural networks have increasingly been incorporated into counterfactual outcome prediction [8]. To this end, we proposed two ML algorithms, GE‐SCORE and GE‐MIMIC, to address limitations of G‐estimation. These two algorithms effectively estimate the causal effect of a time‐varying intervention on a time‐to‐event outcome with the presence of time‐varying confounders, under the SNAFTM framework.

This paper is organized as follows. We first briefly review the SNAFTM and its existing estimation method—G‐estimation. Then, two ML algorithms—GE‐SCORE and GE‐MIMIC—are introduced as the major contribution of this research work. Next, the performances of the existing and proposed methods are compared by simulations. Finally, we apply the proposed algorithms to a real observational health study (Coronary Artery Risk Development in Young Adults Study). The paper is closed with a discussion section, which includes some limitations and future work.

## 2 Method

### 2.1 Notations

In an observational study, visits are scheduled at discrete time points $t_{0},t_{1},\ldots ,t_{K}$ with visit number k=0,1,…,K. Let V be the subject's last visit before the event or the end of his/her follow‐up, whichever comes first. For subject i, i=1,…,N, a risk factor could be a time‐dependent random process with the value at visit k is recorded as $L_{i,k}$, potentially high‐dimensional. Similarly, the exposure level is a random process with values $A_{i,k}$. We assume the risk factor/exposure processes jump at and only at the measured times and A(t) jumps right after L(t). The subject subscript i is suppressed for the following discussion. Vectors are not necessarily in bold fonts.

In this paper, the exposure only takes binary values: $A_{k}=0\;\text{or}\;1$, ${Ā}_{k}=(a_{0},\ldots ,a_{k})$ and ${\bar{L}}_{k}=(l_{0},\ldots ,l_{k})$ record the exposure and covariate histories (realizations) up to the visit k, respectively. Some examples of treatment regimes are “always treated”: Ā=(1,…,1) and “never treated”: Ā=(0,…,0). A dynamic treatment regime is a treatment given at visit k that may depend on covariate histories up to this visit, such as $A_{k}=g({\bar{l}}_{k})$. For simplicity we assume that the variables $L_{k}$ and $A_{k}$ take their values in countable sets, denoted by ${ℒ}_{k}$ and ${𝒜}_{k}$. Lastly, 𝒢 denotes the set of all possible treatment regimes. The treatment regime mapping ${ℒ}_{0}\times \cdots \times {ℒ}_{k}\to {𝒜}_{0}\times \cdots \times {𝒜}_{k}$ is defined by ${\bar{g}}_{k}({\bar{l}}_{k})=(g_{0}(l_{0}),g_{1}({\bar{l}}_{1}),\ldots ,g_{k}({\bar{l}}_{k}))$.

The outcome of interest Y is the time to the first event. Due to right censoring, the resultant observable outcome is T=min(Y,C), where C is the censoring time. Potentially contrary to fact, $Y^{ā}$ denotes the counterfactual outcome that would be observed if subjects follow the treatment regime Ā=ā. The observations are independent and identically distributed (i.i.d.) samples from the distribution of the random vector $\left(T,{\bar{L}}_{K},{Ā}_{K}\right)$.

### 2.2 Sequential Randomization Assumption

Three assumptions are needed to identify the intervention causal effect unbiasedly when time‐varying confounders persist [9]. The assumption most relevant to this application is the Sequential Randomization Assumption (SRA) [9]. This assumption states that the treatment at visit k is randomly assigned with a randomization probability, which may depend on ${Ā}_{k}$ and ${\bar{L}}_{k}$. Importantly, the treatment assignment is independent from the counterfactual outcome, that is, the counterfactual survival time under this treatment regime.

(1) $$A_{k}⫫Y^{ā}|{\bar{L}}_{k}={\bar{l}}_{k},{Ā}_{k-1}={ā}_{k-1},\text{when}\;Y>t_{k}.$$

The required independence will only be examined for subjects who are still free of the endpoint event at each visit. Conceptually, each subject contributes a number ($V_{i}+1$) of records to the final analysis.

For the other two identifiability conditions, Consistency and Positivity, see Appendix A.

An observational study can be conceptualized as a sequentially randomized experiment when these three identifiability conditions hold, except that the probabilities f{A(k)|A(k−1),L(k)} are unknown and must be estimated. However, the study design cannot guarantee the validity of these conditions. Investigators cannot verify these assumptions empirically, but they can collect as many potential confounders as possible to make these assumptions approximately true.

## 3 SNAFTM and G‐Estimation

As the name suggests, the SNAFTM roots from the accelerated failure time (AFT) model. The AFT model conceptualizes subjects' survival times being compressed or expanded by an acceleration factor, which usually relates to some risk factors. In an SNAFTM, a *blip function* [10] $\gamma (y,{\bar{l}}_{k},{ā}_{k})$ is used to quantify the treatment causal effect on the outcome Y of a final brief “blip” of treatment $a_{k}$ in the interval $\left(t_{k},t_{k+1}\right]$. Specifically, a blip function γ is a well‐defined function that satisfies the following relationship:

(2) $$\begin{array}{ll} & P(Y^{{ā}_{k},\bar{0}}>y|{\bar{l}}_{k},{ā}_{k},Y>t_{k})\\ & \;=P(Y^{{ā}_{k-1},\bar{0}}>\gamma_{k}(y,{\bar{l}}_{k},{ā}_{k})|{\bar{l}}_{k},{ā}_{k},Y>t_{k}). \end{array}$$

Applying the blip function to an outcome *subtracts off or removes* the treatment effect of $a_{k}$ on the interval $\left(t_{k-1},t_{k}\right]$, creating a new (counterfactual) outcome that receives no treatment during those same periods [10]. Applying the blip function to $Y^{{ā}_{k},\bar{0}}$ leaves us with $Y^{{ā}_{k-1},\bar{0}}$. Ultimately, the parameter of research interest in an SNAFTM, ψ, is characterized by the blip function. For simplicity, we use $\gamma (y,{\bar{l}}_{k},{ā}_{k};\psi )$, a function of exposure and covariate histories up to visit k, to denote the index blip function for an SNAFTM.

G‐estimation is the state‐of‐art estimation method for SNAFTM [2]. See Appendix B for more details. The fundamental logic of G‐estimation is that the true causal parameter ψ (in the blip function) should yield an unexposed survival (counterfactual) outcome that is independent of the exposure mechanism (i.e., the SRA is true). To perform G‐estimation, one needs a hypothesized blip function (a function of ψ) and the exposure mechanism, both of which may be based on the domain knowledge. G‐estimation searches for the solution of ψ by repetitively calculating the counterfactual survival time for each ψ on a pre‐specified space and performing the independence test between the unexposed survival outcome and the exposure mechanism at each visit k. With the presence of right censoring, the counterfactual survival time and event indicator need to be further refined by artificial censoring [11].

Nevertheless, G‐estimation has some notable drawbacks. First, the G‐estimation requires a “known” blip function, as well as a correct specification of the exposure mechanism $f(A_{k})$. Both are challenging when the number of prognostic factors is large. Furthermore, artificial censoring is required to accommodate the right censoring commonly encountered in survival data. This approach introduces undesirable properties, including non‐smooth estimating equations. As a result, derivative‐based optimization methods are not applicable, and non‐gradient approaches such as grid search must be used to obtain solutions. Due to the computational burden, the causal parameter ψ is usually estimated on a one‐dimensional space. This only allows the causal effect vary for one prognostic factor. These existing obstacles to G‐estimation raise the need to develop more robust, high‐dimensional, friendly estimation algorithms for the SNAFTM.

### 3.1 GE‐SCORE and GE‐MIMIC Algorithms

A Recurrent Neural Network (RNN) is a type of neural network designed to process sequential data by maintaining a hidden state that captures information from previous time steps. There is a natural link between the nested trial, in which one can construct K+1 sub‐models, and the RNN, in which the loss is minimized at each time step (visit). Specifically, to estimate an SNAFTM, the conditional correlation between the counterfactual outcome and the exposure at each time point, given histories before the exposure $A_{k}$, is an ideal loss function for an RNN. In this research work, two NN‐based ML algorithms, GE‐SCORE and GE‐MIMIC, were proposed to tackle the aforementioned limitations of the G‐estimation. The reader shall see later that both GE‐SCORE and GE‐MIMIC can efficiently digest high‐dimensional input data and provide individualized ${\hat{\psi }}_{k}$ estimation.

#### 3.1.1 RNN Predicted Counterfactual Unexposed Survival Time

**FIGURE 2:**
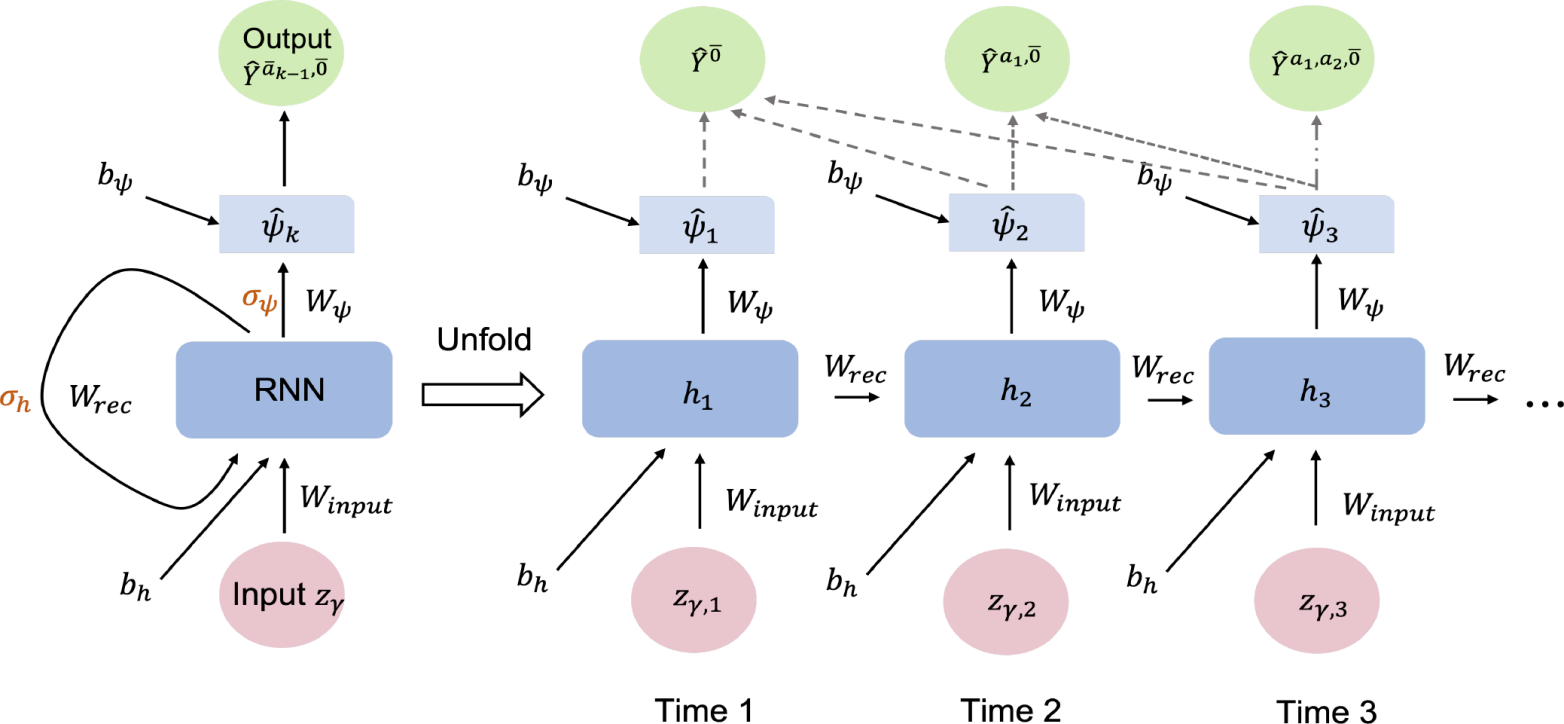
Unfold RNN for the counterfactual unexposed survival time.

In both algorithms, we proposed to use RNN to estimate the sequence of the acceleration factor (i.e., the causal survival ratio) ${\hat{\psi }}_{k}$ (Figure 2). Let $Z_{\gamma }$ be the set of prognostic factors and ${\bar{Z}}_{\gamma ,k}$ denotes their histories. For example, ${\bar{Z}}_{\gamma ,k}$ can include time‐dependent covariate $L_{k}$ such as smoking density (packs/year) collected at each visit k or a static covariate such as gender. At each time k, a hidden state $h_{k}$ is modeled by:

(3) $$h_{k}=\sigma_{h}(W_{\text{rec}}h_{k-1}+W_{\text{input}}Z_{\gamma ,k}+b_{h}),$$

where $Z_{\gamma ,k}$ is the current covariate vector, $h_{k-1}$ is hidden state at the previous step, $W_{\text{input}}$ and $W_{\text{rec}}$ are weights matrices that balance the influence of current input and history on the current hidden state, $b_{h}$ is the bias vector and $\sigma_{h}$ is the activation function for the hidden state.

The predicted acceleration factors reside in a desirable range if an appropriate activation function $\sigma_{\psi }$ of a time‐distributed layer is used:

(4) $${\hat{\psi }}_{k}=\sigma_{\psi }(W_{\psi }h_{k}+b_{\psi }).$$

The overall influence of covariates and their histories on the causal parameter ${\hat{\psi }}_{k}$ is further governed by weights $W_{\psi }$ that are applied to $h_{k-1}$. Supplied with the ${\hat{\psi }}_{k}$ predicted by the RNN unit, we further use an output layer to code the blip function:

(5) $$\gamma_{k}(y,{\bar{l}}_{k},{ā}_{k-1};{\hat{\psi }}_{k})=t_{k}+(y-t_{k})\exp({\hat{\psi }}_{k}a_{k});t_{k}<y\leq t_{k+1}.$$

The RNN thus calculates the counterfactual unexposed survival time backward dynamically, with the time‐varying covariate $Z_{\gamma }$. The final product of the RNN is the sequence of counterfactual unexposed survival time sequence ($Y^{{ā}_{k-1},\bar{0}}$) that assumes the exposure is withheld onward from visit k, calculated by:

(6) $$\begin{array}{l} Y^{{ā}_{k-1},\bar{0}}=t_{k}+\int_{t_{k}}^{Y}\exp\{{\hat{\psi }}_{u}a_{u}\}du. \end{array}$$

Note that ${\hat{\psi }}_{k}$ predicted by the proposed algorithms has a much more extensive meaning compared to a constant ψ estimated by a G‐estimation because it absorbs time‐varying prognostic factors that entered the RNN.

Next, let ${\bar{Z}}_{k}=\{{\bar{L}}_{k},{Ā}_{k-1}\}$ denote all treatment and covariate histories observed prior to $A_{k}$. G‐estimation for the SNAFTM is equivalent to testing the following hypothesis at visit k:

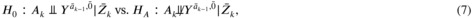

This null hypothesis can be additionally written with respect to a conditional distribution of the treatment regime:

(8) $$H_{0}:Pr(A_{k}|{\bar{Z}}_{k},Y^{{ā}_{k-1},\bar{0}})=Pr(A_{k}|{\bar{Z}}_{k})∼q_{H_{0}}(A_{k}).$$

This conditional independence testing (CIT) problem naturally motivates us to leverage recent advances in neural network‐based CIT methods [12]. By minimizing the loss, which measures the conditional dependence between the unexposed survival time and the exposure mechanism to zero, the RNN would yield counterfactual outcomes that are conditionally independent of the exposure with estimation of the true causal parameter $\psi_{k}^{*}$. However, these two proposed algorithms use different mechanisms to enforce the desirable conditional independence that is stated in Formula (7).

Note that artificial censoring is incorporated into the output layer of the counterfactual outcome network to address the right censoring issue. Define the minimal potential follow‐up as:

(9) $$C({\hat{\psi }}_{k},k)=\min\left\{t_{k}+\overset{K}{\underset{u=k}{\sum }}(t_{u+1}-t_{u})\exp({\hat{\psi }}_{u}a_{u})\right\};k=0,\ldots ,K.$$

This defines the earliest time on the transformed scale at which there could be censoring. Here, the same time scale transformation that is applied to the event time is applied to the potential censoring time. While training the network, the minimum is taken over all empirical $C({\hat{\psi }}_{k},k)$ values for subjects in a batch, recursively at each visit k. We follow [11] to use the empirical censoring value instead of the theoretical value. If subjects have different follow‐up times due to staggered entry, we will use the largest follow‐up time for all subjects in a batch to calculate the minimal potential follow‐up time [2]. These data manipulations are designed to decrease the artificial censoring rate and thus maintain more information.

Once the network produces the counterfactual survival time $H({\hat{\psi }}_{k},k)$, the artificial censoring indicator and artificial censored time follow as

(10) $$X(\hat{\psi },k)=\min(H(\hat{\psi },k),C(\hat{\psi },k))$$

and

(11) $$\Delta (\hat{\psi },k)=1\{H(\hat{\psi },k)<C(\hat{\psi },k)\}$$

As demonstrated in Appendix B, any function of $\left\{X(\hat{\psi },k),\Delta (\hat{\psi },k)\right\}$ is independent of the exposure assigned at time k under the SRA. The existing G‐estimation only employs the artificial censoring indicator as the representative of the counterfactual outcomes in the estimating equation. Nevertheless, the indicator is a step function, which is not preferred when the true parameter space is high‐dimensional. The algorithm often fails to converge to optimal values due to computational difficulties [11]. A suggested remedy is to use a smoothed version of the indicator [11]. Explicitly,

$$\begin{array}{ll} \Delta^{*}(\hat{\psi },k) & =\Delta (\hat{\psi },k)\left\{\frac{C(\hat{\psi },k)-X(\hat{\psi },k)}{C(\hat{\psi },k)}\right\}\\ & =\left\{\begin{array}{ll} 1-\frac{X(\hat{\psi },k)}{C(\hat{\psi },k)}, & \text{if}\;X(\hat{\psi },k)<C(\hat{\psi },k)\\ 0, & \text{if}\;X(\hat{\psi },k)=C(\hat{\psi },k). \end{array}\right. \end{array}$$

Note that $\Delta^{*}(\hat{\psi },k)$ has a range of [0,1] which is more friendly to the optimization problem. This smoothed artificial censoring indicator variable is used in the proposed algorithms. Furthermore, given the fact that the artificial censoring indicator and the time should both be conditionally independent of $A_{k}$ given the exposure and the covariate histories ${\bar{Z}}_{k}$, both could contribute to the estimation. Thus, the proposed algorithms use bi‐variate output $\left\{X(\hat{\psi },k),\Delta^{*}(\hat{\psi },k)\right\}$ enforce the conditional independence. The conditional dependency (i.e., the loss) will be computed between each of $\left\{X(\hat{\psi },k),\Delta^{*}(\hat{\psi },k)\right\}$ and the vector related to the exposure mechanism. The final objective function is the sum of these two losses.

In summary, the contributions of applying RNN in estimating the SNAFTM are two‐fold: (1) allow a rich parameterization of the blip function which reflects the individualized causal effect, (2) improve the estimation accuracy because the SRA is ensured in a more robust manner. The next two sub‐sections present the loss functions of GE‐SCORE and GE‐MIMIC.

#### 3.1.2 GE‐SCORE

**FIGURE 3:**
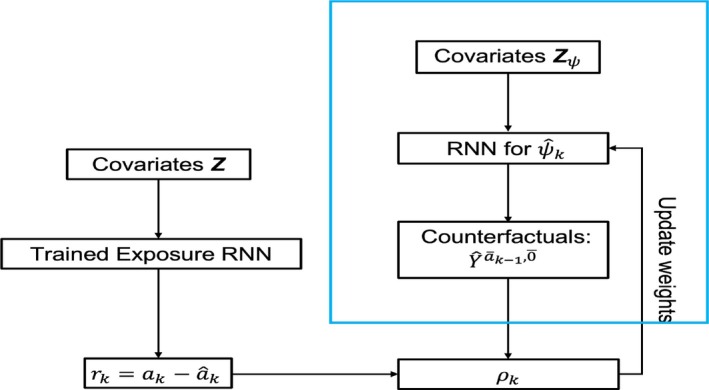
Algorithm flow chart for GE‐SCORE.

In GE‐SCORE, two separate RNNs are used to fit the exposure mechanism $a_{k}$ and the counterfactual outcomes, where the former is trained separately using observed exposure data. Once the exposure mechanism RNN is ready, it is used to calculate the residual $r_{k}$ between the observed $a_{k}$ and the prediction ${â}_{k}$. The weights of the counterfactual outcome RNN will be updated based on the correlation $\rho_{k}$. Figure 3 provides the working flow chart of GE‐SCORE.

Under the null hypothesis, the SRA guarantees that if the exposure model can be correctly specified, $f(A_{k}|{\bar{L}}_{k},{Ā}_{k-1};\alpha_{0})=f(A_{k}|{\bar{Z}}_{k};\alpha_{0})$. Here, $\alpha_{0}\in {\mathbb{R}}^{p}$ is an unknown parameter and, for each $\alpha \in {\mathbb{R}}^{p}$, $f(A_{k}|{\bar{Z}}_{k};\alpha )$ is a density with respect to a measure μ. As introduced in Section 3, θ denotes the coefficient of the extra term of counterfactual outcome to be added to the exposure model, and ψ is the causal parameter of interest, with dimension ν, potentially ν>1. We define the likelihood as:

(12) $$\begin{array}{ll} ℒ(\alpha ,\theta ,\psi ) & =\overset{N}{\underset{i=1}{\prod }}{ℒ}_{i}(\alpha ,\theta ,\psi )\\ & =\overset{N}{\underset{i=1}{\prod }}\overset{V_{i}}{\underset{k=0}{\prod }}{ℒ}_{k,i}(\alpha ,\theta ,\psi )\\ & =\overset{N}{\underset{i=1}{\prod }}\overset{V_{i}}{\underset{k=0}{\prod }}\frac{\begin{array}{l} f(A_{k,i}|{\bar{Z}}_{k,i};\alpha )\\ \cdot \exp[\theta Q_{k,i}\{A_{k,i},H_{i}(\psi ,k)\}] \end{array}}{\begin{array}{l} \int f(a_{k}|{\bar{Z}}_{k,i};\alpha )\\ \cdot \exp[\theta Q_{k,i}\{A_{k,i},H_{i}(\psi ,k)\}]d\mu (a_{k}) \end{array}} \end{array}$$

Here, $\theta \in {\mathbb{R}}^{\nu }$ and $Q_{k,i}\{A_{k,i},H_{i}(\psi ,k)\}=q(A_{k,i},H_{k,i}(\psi ),{\bar{Z}}_{k,i})\in {\mathbb{R}}^{\nu }$, where q() is a fixed function. For computational simplicity, we set $Q_{k,i}\{A_{k,i},H_{i}(\psi ,k)\}=[G_{k,i}\{H_{i}(\psi ,k)\}]A_{k,i}$. Additionally, $G_{k,i}\{H_{i}(\psi ,k)\}=g_{k}(H_{i}(\psi ,k),{\bar{Z}}_{k,i})\in {\mathbb{R}}^{\nu }$, where $g_{k}()$ is a fixed function of $\left\{H_{i}(\psi ,k),{\bar{Z}}_{k,i}\right\}$. For example, if ν=1, $g_{k}(H_{i}(\psi ,k),{\bar{Z}}_{k,i})$ can simply equal to $H_{i}(\psi ,k)$. Given true $f(A_{k}|{\bar{Z}}_{k};\alpha_{0})$, unbiased counterfactual outcomes recovered from Equation (6). Under SRA ${ℒ}_{k,i}(\alpha ,\theta ,\psi^{*})$ is a correctly specified model for $f(A_{k}|{\bar{Z}}_{k},H(\psi^{*}))$ with true values $\alpha_{0}$ and $\theta_{0}=0$. Thus, ${ℒ}_{k,i}(\alpha ,\theta ,\psi^{*})$ is a correctly specified partial likelihood. Let $\tilde{\alpha }=\tilde{\alpha }(\psi )$ maximize ℒ(α,0,ψ) and $\left(\hat{\alpha },\hat{\theta }\right)$ maximize $ℒ(\alpha ,\theta ,\psi^{*})$. Define

(13) $$S_{\theta ,k,i}(\alpha ,\theta ,\psi )=\frac{\partial \log{ℒ}_{k,i}(\alpha ,\theta ,\psi )}{\partial \theta }.$$

Other score functions can be defined analogously. Solving $S_{\theta }(\tilde{\alpha },0,\psi )=0$ leads to a consistent estimation of ψ. Taking the partial derivative of $\log{ℒ}_{k,i}(\alpha ,\theta ,\psi )$ with respect to θ directly leads to

(14) $$\begin{array}{ll} & S_{\theta ,k,i}(\tilde{\alpha },0,\psi )\\ & \;=\frac{\partial \log{ℒ}_{k,i}(\alpha ,\theta ,\psi )}{\partial \theta }\\ & \;=\frac{\partial }{\partial \theta }\left\{\log f(A_{k,i}|{\bar{Z}}_{k,i};\alpha )+\theta Q_{k,i}\{A_{k,i},H_{i}(\psi ,k)\}\right.\\ & \;\left.-\log\int f(a_{k}|{\bar{Z}}_{k,i};\alpha )\cdot \exp[\theta Q_{k,i}\{A_{k,i},H_{i}(\psi ,k)\}]d\mu (a_{k})\right\}\\ & \;=Q_{k,i}\{A_{k,i},H_{i}(\psi ,k)\}\\ & \;-\frac{\begin{array}{l} \int f(A_{k,i}|{\bar{Z}}_{k,i};\alpha )\cdot \exp[\theta Q_{k,i}\{A_{k,i},H_{i}(\psi ,k)\}]\\ \cdot \;Q_{k,i}\{A_{k,i},H_{i}(\psi ,k)\}d\mu (a_{k}) \end{array}}{\int f(A_{k,i}|{\bar{Z}}_{k,i};\alpha )\cdot \exp[\theta Q_{k,i}\{A_{k,i},H_{i}(\psi ,k)\}]d\mu (a_{k})}\\ & \;\overset{\alpha =\tilde{\alpha },\theta =0}{=}Q_{k,i}\{A_{k,i},H_{i}(\psi ,k)\}-\int f(A_{k,i}|{\bar{Z}}_{k,i};\tilde{\alpha })\\ & \;\cdot Q_{k,i}\{A_{k,i},H_{i}(\psi ,k)\}d\mu (a_{k}). \end{array}$$

When ν=1 and we set $G_{k,i}\{H_{i}(\psi ,k)\}=H_{i}(\psi ,k)$, the above score function can further be reduced to

(15) $$\begin{array}{ll} S_{\theta ,k,i}(\tilde{\alpha },0,\psi ) & =H_{i}(\psi ,k)\cdot A_{k,i}\\ & \;-\int f(A_{k,i}|{\bar{Z}}_{k,i};\tilde{\alpha })\cdot H_{i}(\psi ,k)\cdot A_{k,i}d\mu (a_{k})\\ & =H_{i}(\psi ,k)\cdot \left[A_{k,i}-\int f(A_{k,i}|{\bar{Z}}_{k,i};\tilde{\alpha })\cdot A_{k,i}d\mu (a_{k})\right]\\ & =H_{i}(\psi ,k)\cdot [A_{k,i}-E(A_{k,i}|{\bar{Z}}_{k,i})]. \end{array}$$

Last, the loss function for GE‐SCORE is based on the score function:

(16) $$S_{\theta }(\tilde{\alpha },0,\psi )=\overset{N}{\underset{i=1}{\sum }}\overset{V_{i}}{\underset{k=0}{\sum }}S_{\theta ,k,i}(\tilde{\alpha },0,\psi )=0.$$

Solving this score function is equivalent to minimizing the empirical linear correlation between the counterfactual unexposed survival time and the residuals of the exposure model. Borrowing the score function idea, GE‐SCORE uses the Pearson correlation coefficient between the residuals and the counterfactual unexposed survival time as the loss function. A zero Pearson correlation coefficient indicates that adding the counterfactual unexposed time term cannot provide any new information to explain $A_{k}$, if the exposure model already contains ${\bar{Z}}_{k}$.

#### 3.1.3 GE‐MIMIC

In GE‐SCORE, the conditional independence is guaranteed by a zero coefficient before the counterfactual unexposed survival time when it is added to the exposure model as an extra term. However, it is possible that $A_{k}$ and H(k,ψ) are non‐linearly dependent. Thus, more robust algorithms should ensure both linear and non‐linear conditional independence between the counterfactual outcomes and the exposure mechanism. Similar to GE‐SCORE, GE‐MIMIC uses two separate RNNs for the exposure mechanism and the counterfactual outcomes.

**FIGURE 4:**
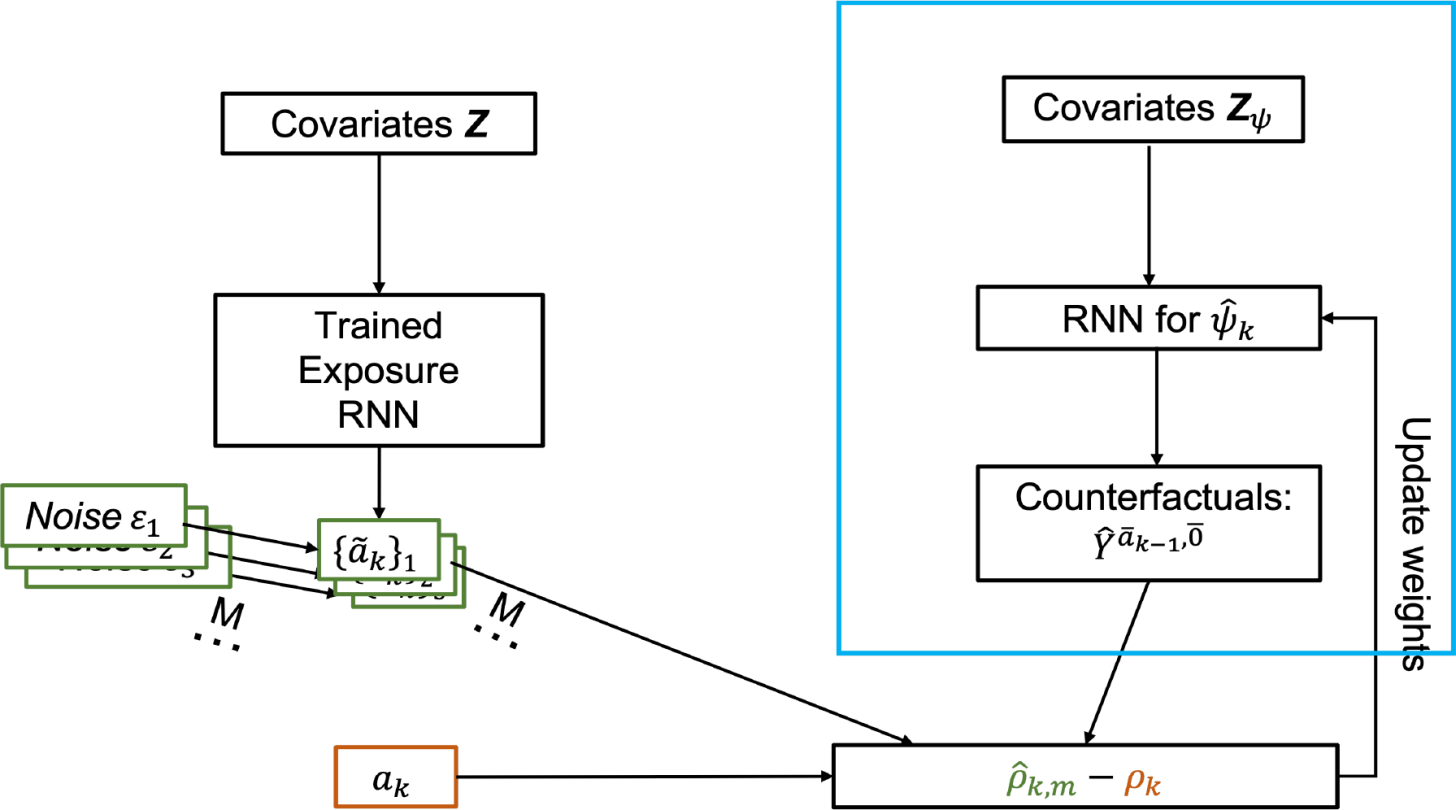
Algorithm flow chart for GE‐MIMIC.

The null hypothesis (conditional independence) is likely to be true when the correlation obtained with observed $a_{k}$ and $Y^{{ā}_{k-1},\bar{0}}$ is close to the same correlation measure obtained under $H_{0}$ (8). Yet, the distribution of exposure under $H_{0}$ is not accessible to researchers. We propose to first generate a number of data sets that are consistent with $H_{0}$, namely, to repeatedly generate ${ã}_{k}$ conditional on ${\bar{z}}_{k}$. Again, ML algorithms such as RNN are powerful tools to fulfill this goal. Then, a statistic is used to capture the dependency between $A_{k}$ and $Y^{{ā}_{k-1},\bar{0}}$. Computing the statistic for each generated copy of ${ã}_{k}$ and $Y^{{ā}_{k-1},\bar{0}}$ will approximate the distribution of the statistics under the null hypothesis. GE‐MIMIC arises from the idea that the true counterfactual should yield similar statistics with either observed $a_{k}$ or hypothesized ${ã}_{k}$ because the latter is generated under the conditional independence assumption. Figure 4 provides the working flow chart of GE‐MIMIC.

Usually, the practitioners have confidence in specifying the null distribution of exposure $Pr(A_{k}|{\bar{Z}}_{k})$. For a discrete exposure variable, the goal is actually to learn the probability of the exposure variable A taking each possible value. As a result, desired null samples ${ã}_{k}$ can be simply generated by repeatedly drawing from the possible outcome set with the learned probability from observed data. It is not surprising to note again that deep networks such as RNN are extremely powerful in predicting the $Pr(A_{k}|{\bar{Z}}_{k})$. The validity of this algorithm is ensured by the exchangeability of random variables [13, 14]. Roughly speaking, under the null hypothesis (8), the generated sequence of random triples $({\left\{{ã}_{k}\right\}}_{m},Y^{{ā}_{k-1},\bar{0}},{\bar{Z}}_{k}),m=1,\ldots ,M$ are exchangeable at each visit k. Consequently, the sequence of summary statistic $\rho_{k,m}$, which is a measurable function of the generated sequences, is also exchangeable. For a specific predicted counterfactual outcome sequence $Y^{{ā}_{k-1},\bar{0}}$, by construction, the resulting random sequence of data sets $({\left\{\tilde{a_{k}}\right\}}_{m},Y^{{ā}_{k-1},\bar{0}},{\bar{z}}_{k}),m=1,\ldots ,M$ is exchangeable at each visit k. This is justified by the fact that ${\left\{{ã}_{k}\right\}}_{m}$ is generated by sampling from the same distribution, at each m. Therefore, the sequence of statistics $\rho_{k,m}$ is also exchangeable at each k.

Next, we argue that the null samples ${ã}_{k}$ generated by the regressor part preserve the correlation between $a_{k}$ and ${\bar{z}}_{k}$ but remove all potential correlations between the counterfactual outcome $Y^{{ā}_{k-1},\bar{0}}$ and $a_{k}$. For the discrete exposure case, a well‐trained prediction algorithm can estimate the conditional distribution $q_{H_{0}}(A_{k})$ (RHS of the null hypothesis (8)) sufficiently. The null samples generated from the null model preserve the dependence structure $Pr(A_{k},{\bar{Z}}_{k})$ but break any dependence between $A_{k}$ and $Y^{{ā}_{k-1},\bar{0}}$. Intuitively, intervention ${Ã}_{k}$ is assigned without looking at the counterfactual outcome $Y^{{ā}_{k-1},\bar{0}}$ at each visit k. Replacing the $A_{k}$ with ${Ã}_{k}$ will break the causal path from $Y^{{ā}_{k-1},\bar{0}}$ to $A_{k}$ if there is any. Per the law of large numbers, the $\rho_{k}$ should be close to the mean of ${\hat{\rho }}_{k,m}$ under the null hypothesis when M is large. Thus, we propose to use the absolute difference between the mean of ${\hat{\rho }}_{k,m}$ and the observed $\rho_{k}$ as the loss to be minimized.

To regularize the prediction, we considered adding the variance of ${\hat{\psi }}_{k}$ at each k as a penalty term in the loss function. It directly penalizes the prediction complexity, thus the predicted values are encouraged to be less variant. With increasing weight of the penalty term in the total loss, the algorithm is forced to generate less variant, even constant output for ${\hat{\psi }}_{k}$. The algorithms thus have the potential to reduce to the one‐dimensional G‐estimation. Thus, the choice of weights of the penalty is a delicate question. In GE‐SCORE, the objective function is the absolute value of the correlation coefficient with a range of [0,1]. In GE‐MIMIC, ρ lies between −1 and 1 and the absolute difference between ρ and $\hat{\rho }$ is expected to be small. To increase the gradients returned at each optimization iteration, we also magnified the loss in both algorithms by 100. As a result, the theoretical range of GE‐SCORE loss is [0,100]. For GE‐MIMIC, the range is even broader. Now with the penalty term, the loss function for GE‐SCORE at visit k is:

(17) $${ℒ}_{GE-SCORE,k}^{\prime }=\gamma_{1}\cdot {ℒ}_{GE-SCORE,k}+\gamma_{2}\cdot \mathrm{var}({\hat{\psi }}_{k}).$$

Similarly, the loss function for GE‐MIMIC at visit k is:

(18) $${ℒ}_{GE-MIMIC,k}^{\prime }=\gamma_{1}\cdot {ℒ}_{GE-MIMIC,k}+\gamma_{2}\cdot \mathrm{var}({\hat{\psi }}_{k}).$$

In both loss functions, $\gamma_{1}$ and $\gamma_{2}$ control the contribution of each term to the total loss. Per our previous discussion, $\gamma_{1}=100$. After some experiments, we found that adding the penalty term with $\gamma_{2}=10$ worked well. These parameters are used in all following simulations and applications, but are subject to change based on the belief of a strong homogeneity.

Additional parameter tuning for these algorithms was discussed in the Appendix C.

## 4 Simulation

### 4.1 Simulation Setting

The simulation mostly follows a well‐accepted literature [15] on generating time‐varying confounders. The hypothetical study has three visits k=(0,1,2), scheduled at year $t_{k}=(0,2,4)$. Subjects (N=3000) are followed for an additional two years after the last visit, hence the maximal follow‐up time $t_{K}=6$. To reflect the diverse biological nature of distinct disease processes and to consider that the true causal effect can be either beneficial or adverse, the following scenarios for the blip function (which) were evaluated:

- Simulation Scenario I. The true exposure causal effect is a constant (0.5 or −0.5) across all subjects and all time points.
- Simulation Scenario II. The true exposure causal effect at each time point k is modified by another covariate (e.g., $L_{k}$). The true causal effect is: $\psi_{k}^{*}=\psi_{1}+\psi_{2}L_{k}$. The simulated causal effect ranges from −0.4 to 1 (beneficial case) and −1 to 0.4 (adverse case).
- Simulation Scenario III. The true exposure causal effect further relates to exposure and covariate histories. The corresponding true causal effect at baseline is $\psi_{0}^{*}=\psi_{1}+\psi_{2}\text{Gender}+\psi_{3}L_{1}+\psi_{4}\text{Gender}\times L_{1}$. This reflects that the causal exposure effect is modified by the baseline characteristic gender, $L_{1}$, as well as an interaction between these two. For k>0, $\psi_{k}^{*}=\psi_{1}+\psi_{2}\text{Gender}+\psi_{3}\sum_{t=1}^{k}\left(\frac{1}{t}{\bar{L}}_{k-t}{Ā}_{k-t}\right)+\psi_{3}L_{k}+\psi_{4}\text{Gender}\times L_{k}$. An interaction term $L_{k}A_{k}$ equals zero if one is never exposed, but accumulates over time if continuously exposed. The factor $\frac{1}{t}$ controls the magnitude of the histories; previous exposure farther back in time has a smaller, if any, influence on the causal effect of current exposure. The simulated causal effect ranges from −0.3 to 1.5 (beneficial case) and −1.5 to 0.3 (adverse case).
  Scenario III becomes plausible when the true exposure effect relates to exposure history, as well as an effect modifier in the present. For instance, it is obvious that the current smoking causal effects differ between a smoker who has been smoking for the past 10 years and another smoker who just started last month.
- Simulation Scenario IV. The true exposure causal effect is a nonlinear function of gender, L, and an interaction between them:
(19) $$\psi_{k}^{*}=f(x_{t})=-\log\left[\frac{3}{1+\exp(-x_{t}^{2})}\right].$$
  The $x_{t}$ is calculated per time point: $x_{0}=\psi_{1}+\psi_{2}\text{Gender}+\psi_{3}L_{1}+\psi_{4}\text{Gender}\times L_{1}$, when k>0, $x_{k}=\psi_{1}+\psi_{2}\text{Gender}+\psi_{3}\sum_{t=1}^{k}\left(\frac{1}{t}{\bar{L}}_{k-t}{Ā}_{k-t}\right)+\psi_{3}L_{k}+\psi_{4}\text{Gender}\times L_{k}$. The nonlinear function (19) indicates that the exposure causal effect increases whenever the $x_{t}$ diverges from 0, yet caps at a certain value. The simulated causal effect ranges from 0.4 to 1.1. The case of adverse exposure causal effect is modeled by removing the negative sign before log and ranges from −1.1 to −0.4.

For a beneficial causal effect, the censoring rate is around 40%, while for a harmful causal effect, the censoring rate is around 20%. The exposure mechanism can be as simple as a logistic regression or follow a more complicated decision rule. Methods that estimate SNAFTM we compared include GE‐SCORE and GE‐MIMIC with G‐estimation. Another important class of models that adjusts for time‐varying confounders—Marginal Structural Model (MSM) [16] was also explored to compare with the SNAFTM. See Appendix D for more details of the simulation. Each scenario was repeated 100 times.

### 4.2 Simulation Results

**FIGURE 5:**
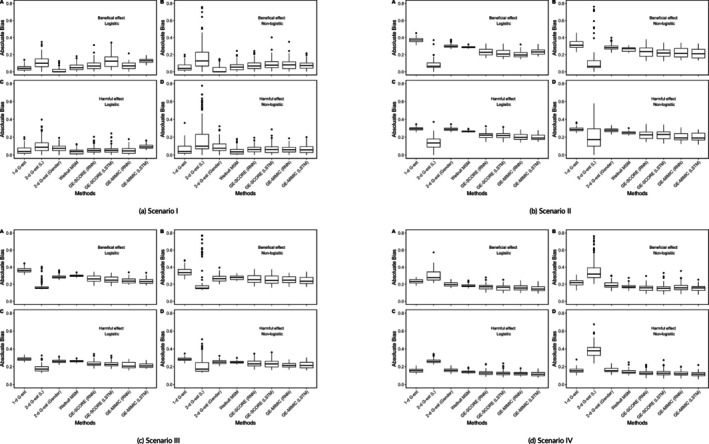
Simulation results: Box plots of bias of estimated causal effects across different models. (a) Scenario I, (b) Scenario II, (c) Scenario III, and (d) Scenario IV.

Figure 5 presents the distribution of the absolute estimation bias of 100 repetitions, under four simulation scenarios.

For Scenario I (Figure 5a), the 1‐*d* G‐estimation performed well because this is the “correct” model for a constant acceleration factor. The Weibull MSM yielded very similar results compared to the 1‐*d* G‐estimation. The 2‐*d* G‐estimation also seems reasonable when the blip function contains a low‐dimensional discrete covariate (i.e., gender), given is still searched on a low‐dimensional space. However, the estimation bias significantly increased when a wrong covariate (i.e., continuous $L_{k}$) entered the blip function specification. On the other hand, though GE‐SCORE and GE‐MIMIC both search on a high‐dimensional space, their performance is close to the benchmark 1‐*d* G‐estimation with slightly more variant predictions. For this simplest case, using an over‐complicated recurrent unit such as the LSTM brought no benefits but more bias.

Scenario II is slightly more complicated, in which the exposure causal effect is no longer a constant but depends on an individualized prognostic factor $L_{k}$. Figure 5b showed that the performance of G‐estimation surpasses MSM because G‐estimation can account for heterogeneous causal effects. However, G‐estimation only succeeds when the true treatment effect modifier $L_{k}$ is correctly included in the blip function. GE‐SCORE and GE‐MIMIC cannot outperform G‐estimation when the blip function is correctly specified, but these algorithms are more efficient than the 1‐*d* G‐estimation and more robust to different exposure mechanisms. Starting from this scenario, using a more complicated recurrent unit such as LSTM in GE‐SCORE and GE‐MIMIC is beneficial because it helps the algorithms capture the increasingly complex connected nature of covariates and exposure causal effect.

Figure 5c also shows that 1‐*d* G‐estimation failed to address scenario III. On the other hand, 2‐*d* G‐estimation with $L_{k}$ as the covariate may succeed when the exposure mechanism is correctly specified because $L_{k}$ is the dominant effect modifier for the exposure causal effect. However, the estimation exhibited substantial variability when the exposure mechanism is mis‐specified, indicating that the resulting values are unstable and should not be interpreted with confidence. On the other hand, GE‐SCORE and GE‐MIMIC keep performing stably. They have a similar performance as the 2‐*d* G‐estimation, but the variance of bias is smaller. Between these two algorithms, GE‐MIMIC is more appealing under this scenario because it is expected to incorporate a more sophisticated time‐connected intervention causal effect. Furthermore, using LSTM has benefits over simple RNN due to the same reason.

Lastly, scenario IV (Figure 5d) showed that GE‐SCORE and GE‐MIMIC were significantly better than all other methods examined when the risk factors do not affect the exposure causal effect in a linear manner. Even with only one hidden‐layer network, these algorithms can estimate the non‐linear causal effect adequately. Furthermore, the benefits of using a more complicated recurrent unit (LSTM vs. RNN) emerged under this scenario.

Note that in all simulation scenarios, GE‐SCORE and GE‐MIMIC intake the same set of covariates with the same network structure. Although the true causal effect and the exposure model vary dramatically between scenarios (linear vs. nonlinear, related to risk factor L or not, etc.), GE‐SCORE and GE‐MIMIC can robustly estimate the exposure causal effect without specifying the blip function or the exposure mechanism. Though results are not shown here, if the neural network were provided with the correct blip function specifications (e.g., constant input for scenario I), the algorithms outperformed the benchmark G‐estimation.

## 5 Application to CARDIA

The Coronary Artery Risk Development in Young Adults (CARDIA) Study [17, 18] examined the development and determinants of clinical and subclinical cardiovascular disease and their risk factors. This study is a population‐based observational study of 5115 participants aged 18–30 years recruited during 1985–1986, from four centers in the United States. Our research question is the causal effect of smoking (A) on the time to the subject's first occurrence of a CVD event (Y). In such a long‐term observational study, the time‐varying confounding issue is non‐ignorable (Figure 1).

The CARDIA study has high‐quality data, giving a satisfying retention rate and great compliance of subjects. The outcome events of interest—any fatal or non‐fatal CVD event—were observed for 355(6.94%) subjects as of December 31, 2021. Hence, most subjects are right‐censored. Given the long follow‐up range, the staggered entry during the first 1–2 years, and the slightly different individual visit schedules were ignored to ease the computation. Specifically, we use a standard visit schedule at months (0,24,60,84,120,180,240,300,360) for all subjects and use 425 months as the end of study time. Records after the first event were discarded from the analysis. Inverse Probability of Censoring Weights (IPCW) was used to adjust for potentially informative censoring (i.e., death and loss to follow‐up). After necessary data cleaning, there were 5111 records available for the baseline models and 39 671 records available for the time‐dependent models.

First, traditional statistical analysis methods identified a passive effect of smoking on the time to first CVD event (Table 1). The time‐dependent models identified a significantly more harmful effect of smoking compared to the baseline models.

**TABLE 1:** Estimated hazard ratio of current smokers vs. non‐smokers on time‐to‐first CVD using traditional survival models that do not adjust for time‐varying confounders, CARDIA.

Model	Estimated hazard ratio (95% confidence interval)
Cox PH	2.27 (1.80, 2.89)
Time‐dependent Cox PH	2.42 (1.88, 3.11)
Weibull PH	2.27 (1.82, 2.84)
Time‐dependent Weibull PH	2.50 (2.05, 3.05)

*Note:* All models adjusted for gender (female vs. male), race (white vs. non‐white), age at enrollment (younger than 18, 19–25, 25–29, and older than 30), marital status (married or living as married, divorced or separated or widowed, never married), education level (less than high school or GED, some college or college, college above), employment status (full‐time/part‐time/unemployed), BMI, SBP, self‐reported diabetic/hypertension/high cholesterol, exercise intensity score, whether the subject is a drinker or not and family history of heart disease (whether father/mother had a heart attack and whether father/mother had a stroke).

To address the time‐varying confounding issue, we now consider an SNAFTM:

(20) $$S_{Y^{{ā}_{k-1},\bar{0}}|{\bar{L}}_{k}={\bar{l}}_{k},{Ā}_{k}={ā}_{k},Y>t_{k}}\{\gamma_{k}(y,{\bar{l}}_{k},{ā}_{k};\psi )\}=S_{Y^{{ā}_{k},\bar{0}}|{\bar{L}}_{k}={\bar{l}}_{k},{Ā}_{k}={ā}_{k},Y>t_{k}}(y).$$

with the blip function

(21) $$\gamma_{k}(y,{\bar{l}}_{k},{ā}_{k};\psi )=t_{k}+(y-t_{k})\exp(\psi a_{k}).$$

This model didn't consider any effect modifiers for the causal effect. Thus, it assumed the smoking causal effect is a constant for all subjects across time points. Covariates considered in the exposure model included time‐independent variables: Gender (female vs. male), race (white vs. non‐white), age at enrollment (younger than 18, 19–25, 25–29, and older than 30); time‐varying variables: BMI, education level (less than high school or GED, some college or college, college above), working status (employed full‐time/part‐time/unemployed), marital status (married or living as married/divorced or separated or widowed/never married), self‐reported diabetes/hypertension/high cholesterol, SBP, depression score, exercise intensity score, and alcohol consumption status (drinker or not). The G‐estimation with grid search on range [−1,1] by a step size of 0.01 estimated ψ=0.61 with a confidence interval (0.52,0.72). Thus, the G‐estimated SNAFTM suggested that smoking compressed the CVD‐free time by a factor of exp(−0.61)=0.54 times as of the nonsmoker's CVD event‐free time.

To compare with models that did not adjust for time‐varying confounders, the G‐estimated acceleration factor exp(−ψ) can be related to a rate ratio if one further assumes that the counterfactual unexposed survival time $Y^{\bar{0}}$ follows a Weibull distribution [3, 19]. The resultant smoking causal rate ratio of the CARDIA study is exp((2.25)(0.61))=3.95 with a 95% confidence interval (3.22;5.05), which is significantly larger compared to traditional models (Table 1). Weibull MSM estimated an acceleration rate 0.66(0.61;0.72) with a corresponding causal rate ratio 2.86(2.32;3.52). This MSM estimated causal rate ratio is also larger than the traditional Weibull model (Table 1); however, is much smaller than the G‐estimated accelerated rate. Using a Cox MSM yields a similar estimated causal rate ratio: 2.77(2.16;3.55). The proposed GE‐SCORE and GE‐MIMIC were also explored. The counterfactual outcome network in both algorithms considered six effect modifiers: Smoking status at the previous visit, sex (male vs. female), alcohol drinking status (drinker vs. non‐drinker), smoking intensity (number of cigarettes smoked per day), BMI, and SBP. Data were split into training (70%), validating (20%), and the final prediction was made with respect to 10% test data.

Table 2 compared the acceleration factor estimated by the baseline Weibull model, time‐dependent Weibull, Weibull MSM, G‐estimation, GE‐SCORE, and GE‐MIMIC. GE‐SCORE and GE‐MIMIC estimated individualized acceleration factors; thus, the point estimation mean and range were presented. Using CARDIA data, all models identified a significant negative effect of smoking on the time‐to‐first CVD event. The models that adjusted for time‐varying covariates were more powerful in detecting such a harmful effect. Among all models compared, G‐estimation detected the most significant smoking causal effect, followed by the GE‐MIMIC and GE‐SCORE. Yet, one of the significant benefits of GE‐SCORE and GE‐MIMIC is that they make individual predictions at all investigated visits, which enables precise and personalized health advice. Results of GE‐SCORE and GE‐MIMIC in Table 2 showed that the smoking causal effect does not vary significantly across follow‐up years (aging), although a slightly increasing time trend was observed. The baseline smoking effect seems the most significant and concentrated, not only due to this visit having the largest available data size, but also having the highest proportion of smokers among all follow‐up visits, in this population. Overall, the smoking effect estimated by GE‐MIMIC is more significant compared to GE‐SCORE, with slightly wider ranges.

**TABLE 2:** Estimated smoking effect (acceleration factor) on time to subjects' first CVD event estimated by traditional survival models, CARDIA.

Models	ψ Estimation	Confidence interval
Baseline Weibull	−0.34	(−0.44, −0.24)
Time‐dependent Weibull	−0.45	(−0.56, −0.34)
Weibull MSM	−0.42	(−0.50, −0.33)
G‐estimation	−0.61	(−0.72, −0.52)
GE‐SCORE^a^		
Overall	−0.47	(−0.49, −0.43)
Year 00	−0.48	(−0.49, −0.48)
Year 02	−0.47	(−0.49, −0.45)
Year 05	−0.47	(−0.49, −0.44)
Year 07	−0.47	(−0.49, −0.44)
Year 10	−0.47	(−0.49, −0.44)
Year 15	−0.47	(−0.49, −0.43)
Year 20	−0.47	(−0.49, −0.43)
Year 25	−0.47	(−0.49, −0.43)
Year 30	−0.47	(−0.49, −0.43)
GE‐MIMIC^a^		
Overall	−0.53	(−0.58, −0.49)
Year 00	−0.54	(−0.58, −0.52)
Year 02	−0.53	(−0.58, −0.50)
Year 05	−0.53	(−0.58, −0.50)
Year 07	−0.53	(−0.58, −0.49)
Year 10	−0.53	(−0.58, −0.49)
Year 15	−0.53	(−0.58, −0.49)
Year 20	−0.53	(−0.58, −0.49)
Year 25	−0.53	(−0.58, −0.49)
Year 30	−0.53	(−0.58, −0.49)

^a^ The estimation mean and range were reported. The signs of estimation from different methods have been manually unified to facilitate the comparison. The negative estimations indicate the deceleration of the event‐free time, as in a usual AFT model.

**FIGURE 6:**
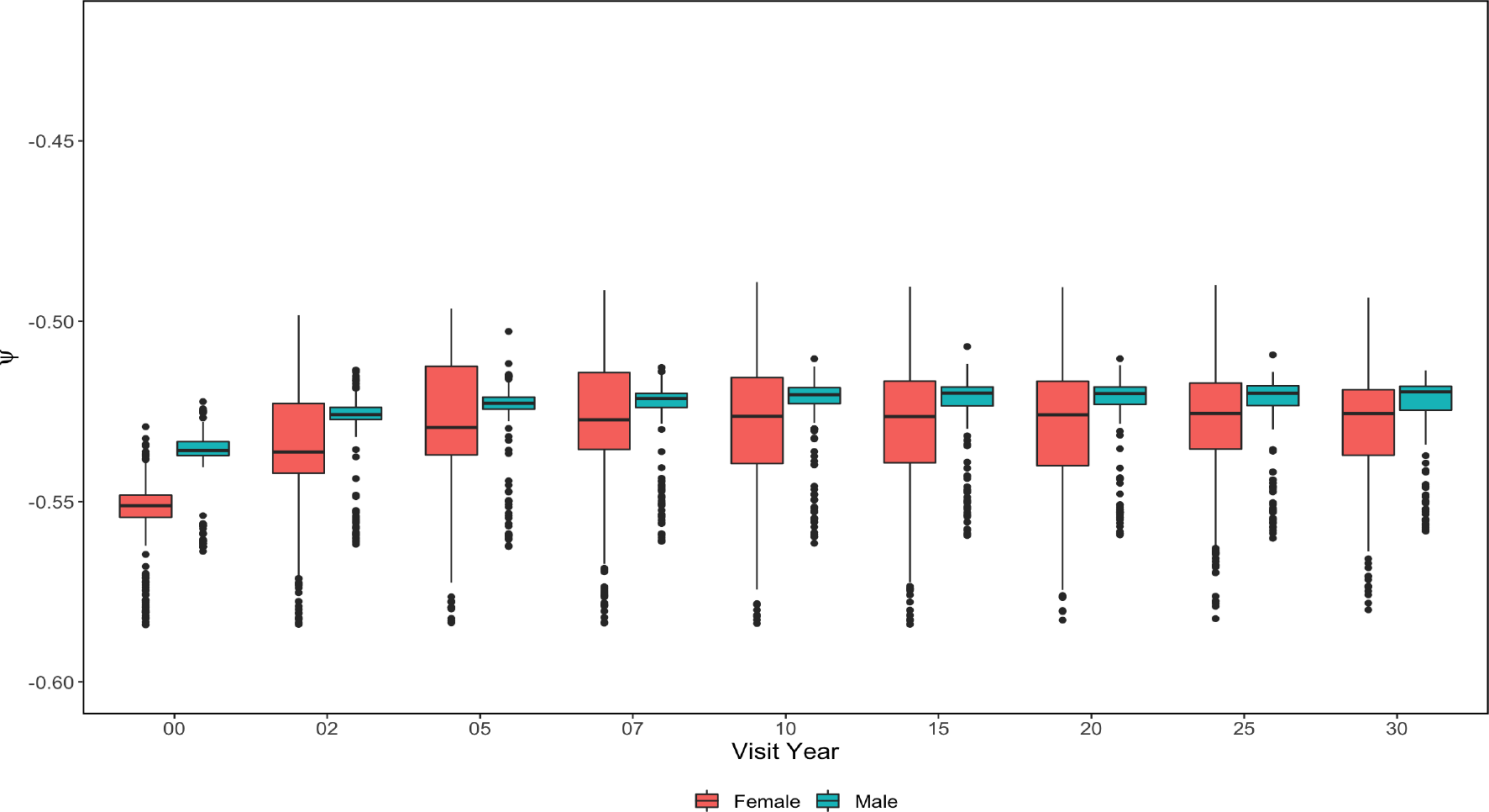
Estimated ${\hat{\psi }}_{k}$ by gender and visit—CARDIA study.

**FIGURE 7:**
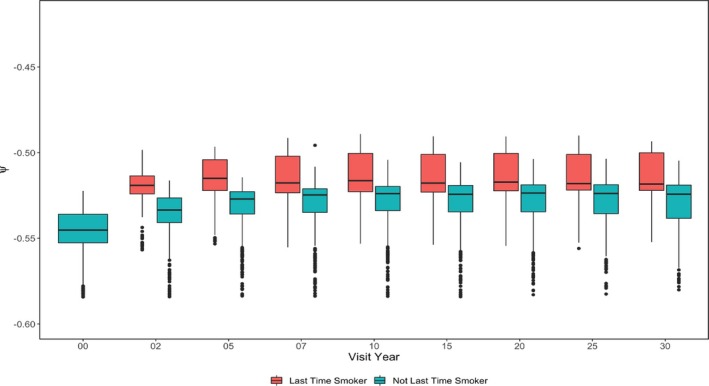
Estimated ${\hat{\psi }}_{k}$ by smoking status at previous visit and visit—CARDIA study.

**FIGURE 8:**
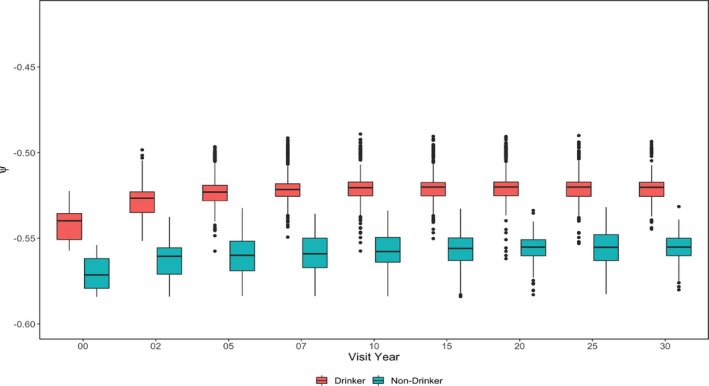
Estimated ${\hat{\psi }}_{k}$ by drinker status and visit—CARDIA study.

For subgroup heterogeneity, we focused on assessing the group‐wise difference for estimated smoking effects $\hat{\psi_{k}}$ by categorical variables considered in the counterfactual network: Gender (Figure 6), smoking status at the previous visit (Figure 7), and drinker status (Figure 8). Interestingly, smoking seems to have a more substantial harmful causal effect on females compared to males. The gender disparity of the smoking effect has been noticed by previous researchers [20]. Also, the estimated smoking causal effect for females has a larger variation, possibly due to a smaller proportion of smokers and a smaller proportion of subjects who experience the event of interest in the female group compared to the male group. Furthermore, the female group may have larger heterogeneities among other important risk factors for CVD events, which can also increase the variation of the estimation. GE‐SCORE didn't identify a significant difference in the smoking causal effect between drinkers and non‐drinkers. However, GE‐MIMIC suggested that smoking is less harmful for drinkers. One possible explanation is that the proportion of drinkers is much higher than that of non‐drinkers in the CARDIA population. We may oversimplify the problem by classifying subjects as a drinker or not a drinker without controlling for the drinking intensity. When considering drinking intensity, a U‐shaped drinking effect on CVD event is most plausible [21]. When considering the smoking status at the previous visit, a new smoker or a relapsed smoker suffers more from current smoking. The conclusion that can be delivered to the public and patients is that never starting smoking, or having stopped, don't start again.

## 6 Discussion

It is well‐known that the hazard rate lacks causal meanings [22, 23]. On the other hand, the SNAFTM estimates the causal survival time acceleration rate effectively while adjusting for the time‐varying confounders. However, its coupled cumbersome G‐estimation method seriously limited its application. This work extended the SNAFTM to the deep learning field by proposing two innovative RNN‐based estimation algorithms—GE‐SCORE and GE‐MIMIC. The proposed algorithms greatly ease the estimation of an SNAFTM and generally outperform the existing G‐estimation. First, these algorithms structure the most plausible blip function by learning from the data. These algorithms can learn arbitrarily complex, non‐linear relationships between inputs and the causal effect without any linearity assumptions baked into the blip function. This self‐learning feature no longer requests a pre‐specified (and usually erroneous) function blip function from the user. Similarly, the need to specify the correct exposure mechanism is greatly reduced because these algorithms could also learn the exposure mechanism from data. Second, the proposed algorithms are able to incorporate heterogeneity when estimating the individual intervention causal effects ${\hat{\psi }}_{k,i}$. This is particularly useful given that the intervention causal effect usually correlates with biological covariates. The proposed algorithms can automatically learn relevant features and interactions from raw data, removing the need for extensive feature engineering or manual selection. Thus, the proposed algorithms provide more valuable information to individuals. Last, the proposed algorithms can handle high‐dimensional inputs for the blip function, while previous G‐estimation relies on an infeasible grid search.

Though not discussed here, the expansion to the non‐binary exposure A is straightforward [2]. Similar ideas can be implemented in GE‐SCORE. For GE‐MIMIC, the MIMIC step aims at approximating the empirical distribution appropriately. As a result, adding some continuous noises to the conditional expectation given histories 𝔼 $\left(A_{k}|{\bar{Z}}_{k}\right)$ [24] approximate the continuous exposure distribution under the null. One may also consider a more advanced null sample generation mechanism (e.g., using a trained Recurrent Conditional Generative Adversarial Net [25]) if over‐complicated exposure mechanisms are anticipated. Nevertheless, the proposed algorithms have much higher chances to succeed in SNAFTM estimation because they are more likely to capture the true intervention mechanism compared to the traditional parametric statistical models, especially when the input covariates for the exposure mechanism is ar a high‐dimensional space.

When comparing the two proposed algorithms, GE‐SCORE directly minimizes the dependency between the counterfactuals and residuals of the intervention model. This algorithm is more intuitive and converges more quickly. But as the complexity of the targeted causal effect increases, the GE‐SCORE's performance deteriorates, as shown in the simulation. GE‐MIMIC is more general in the sense that the desirable conditional independence is not enforced by a zero correlation coefficient between counterfactuals and residuals, but by the similarity of correlation between the observed samples and the generated null samples. The nonlinear dependence can also be captured if a more comprehensive correlation metric (e.g., distance correlation) is used. This extension was explored but not presented in this article because it significantly increased optimization time. Notably, there is a trade‐off between the complexity of the loss function and the generality of the correlation metric. For our explored scenarios, the Pearson correlation coefficient seems sufficient. Furthermore, GE‐MIMIC requires extra time to generate (M) batches of null samples as its first step. While the current approach requires substantial computational power, this challenge is expected to lessen as hardware and algorithmic advances continue.

The proposed algorithms have some important limitations. Losses of both algorithms heavily rely on the correlation coefficient, which may only reflect a trend (either linear or nonlinear) between two random variables. In future work, using a more comprehensive correlation measure, such as distance correlation, may address this issue. The intermediate output of the algorithm—the counterfactual survival time and the causal estimand ${\hat{\psi }}_{k}$ may not be uniquely identified. A possible remedy is to extract more information from unexposed samples in the population. By the consistency assumption, the counterfactual unexposed time is the observed time if the subjects stay unexposed. In conclusion, without a pre‐specified blip function format, learning the causal estimand ${\hat{\psi }}_{k}$ is a challenging problem, especially in the high‐dimensional case. Though a complete causal diagram (e.g., Figure 1) that identifies the causal relationships among all related covariates and the outcome of interest is required to estimate the causal effect of interest, the identification of the causal DAG falls out of the scope of this work.

## 7 Conclusion

The causal inference for the survival outcome is extra challenging when the time‐varying confounding issue persists, though it often attracts researchers' attention. The SNAFTM has been shown to be an effective candidate for this causal problem. Yet, this model is much unpopular due to the computational challenges and implementation difficulties associated with its existing estimation method—G‐estimation. In this work, we aim to proactively address these challenges by proposing two innovative neural network‐based algorithms—GE‐SCORE and GE‐MIMIC to estimate the SNAFTM. These algorithms have appealing features compared to G‐estimation, such as the capability to handle high‐dimensional data, the non‐requirement of a prespecified parametric blip function or an exposure model, and the robustness to different intervention mechanisms. The intermediate output of these algorithms also has clinical meanings. This side product can be interpreted as a factor by which the intervention expands or compresses the event‐free time, similar to the acceleration factor in an AFT model. Under various clinical settings, this factor is easy to communicate with patients, further allowing clinicians to develop personalized treatment plans that adapt to individuals' needs. By using simulated and real clinical data, we showed that with moderate data processing, these algorithms are flexible enough to provide clinically meaningful and individualized causal estimation for the intervention of research interest. In conclusion, GE‐SCORE and GE‐MIMIC would greatly facilitate the application of the SNAFTM, especially in the era of machine learning.

## Funding

The authors have nothing to report.

## Disclosure

The authors have nothing to report.

## Conflicts of Interest

The authors declare no conflicts of interest.

## Data Availability

The data that support the findings of this study are openly available in BioLINCC at: https://biolincc.nhlbi.nih.gov/home/.

## Appendix A Identifiability Conditions

The Consistency assumption states that, for any individual,

(A1)
$$\text{if}\;Ā=ā\;\text{then}\;Y^{Ā}=Y^{ā}=Y.$$

The Positivity assumption states that, for all possible levels of $a_{k}$,

(A2)
$$\begin{array}{ll} & \text{if}\;f({\bar{L}}_{k}={\bar{l}}_{k},{Ā}_{k-1}={ā}_{k-1})>0,\\ & \;\text{then}\;f(a_{k}|{\bar{L}}_{k}={\bar{l}}_{k},{Ā}_{k-1}={ā}_{k-1})>0. \end{array}$$

For dynamic regimes, the positivity assumption stays the same. The SRA is strengthened, for any k, treatment regime g∈𝒢 and covariates history ${\bar{l}}_{k}$,

(A3)
$$A_{k}⫫Y^{g}|{\bar{L}}_{k}={\bar{l}}_{k},{Ā}_{k-1}=g_{k}({\bar{L}}_{k-1},{Ā}_{k-2}),\text{when}\;Y>t_{k}.$$

The consistency assumption is also strengthened. For any k and treatment regime g∈𝒢 and covariates history ${\bar{l}}_{k}$,

(A4)
$$\text{if}\;A_{k}=g_{k}({\bar{L}}_{k},{Ā}_{k-1})\text{, then}\;Y^{g}=Y\;\text{and}\;{\bar{L}}_{K}^{g}={\bar{L}}_{K},$$

where ${\bar{L}}_{k}^{g}$ is the counterfactual L‐history through visit k under regime g.

## Appendix B More Details on SNAFTM and G‐Estimation

Formally, the SNAFTM maps percentiles y of the conditional distribution of the failure time $Y^{{ā}_{k},\bar{0}}$, into $\gamma_{k}(y,{\bar{l}}_{k},{ā}_{k};\psi )$ percentiles of the conditional distribution of $Y^{{ā}_{k-1},\bar{0}}$, given ${\bar{L}}_{k}={\bar{l}}_{k}$ and ${Ā}_{k}={ā}_{k}$, and for subjects who are still at risk (say, alive) at time $t_{k}$ [26]:

(B1)
$$S_{Y^{{ā}_{k-1},\bar{0}}|{\bar{L}}_{k}={\bar{l}}_{k},{Ā}_{k}={ā}_{k},Y>t_{k}}\{\gamma_{k}(y,{\bar{l}}_{k},{ā}_{k};\psi )\}=S_{Y^{{ā}_{k},\bar{0}}|{\bar{L}}_{k}={\bar{l}}_{k},{Ā}_{k}={ā}_{k},Y>t_{k}}(y).$$

Applying the blip function to the failure time outcome Y removes the effect of a final blip of treatment of magnitude $a_{k}$ at visit k on the survival outcome. From the observed time $Y^{a_{k}}$, one can reach $Y^{\bar{0}}$—the “treatment‐free” counterfactual by applying the blip function recursively until all treatment effects are removed. The blip function enables us to quantify the treatment causal effect on the outcome with a few parameters. A simple example blip function is

(B2)
$$\gamma_{k}(y,{\bar{l}}_{k},{ā}_{k};\psi )=t_{k}+\int_{t_{k}}^{y}\exp(\psi a(u))du,$$

in which parameter ψ quantifies the causal survival time ratio between two counterfactual survival outcomes on the log scale, similar to the AFT model. In this example, the treatment causal effect is a constant for the population, regardless of different characteristics. A more complicated blip function can be $\gamma_{k}(y,{\bar{l}}_{k},{ā}_{k};\psi_{1},\psi_{2})=t_{k}+\int_{t_{k}}^{y}\exp((\psi_{1}+\psi_{2}l(u))a(u))du$. This blip function hypothesized an interaction effect between the covariate L and treatment at visit k. Consequently, the null treatment effect is now encoded as $\psi_{1}=\psi_{2}=0$. Based on the true underlying causal relationship, the causal parameter of interest ψ can be a vector taking values on ${\mathbb{R}}^{\nu }$. There is no effect of final blip of treatment of size $a_{k}$ among subjects with observed history ${\bar{l}}_{k},{ā}_{k}$ (i.e., $\gamma_{k}(y,{\bar{l}}_{k},{ā}_{k};\psi )=y$) if and only if the distribution of Y is the same under any treatment regime g [10]. Thus, the treatment causal effect can be tested via a ν dimensional statistical test of ψ=0. Evidently, the example blip functions presented here are simplified versions (e.g., using an identity link function) of the RNNs proposed under section 2.4.

To estimate the causal parameter ψ in the SNAFTM using G‐estimation, one should start with recovering the true unexposed counterfactual variable $Y^{\bar{0}}$ from the observed quantities. Provided with a blip function, $Y^{\bar{0}}$ is a function of ψ. Function H(ψ) is used to make the relationship between $Y^{\bar{0}}$ and ψ explicitly. As an example, under the SNAFTM indexed by the blip function specified by Formula (B2), the counterfactual unexposed survival time can be calculated as:

(B3)
$$Y^{\bar{0}}=H(\psi )=\int_{0}^{Y}\exp\{\psi A(u)\}du.$$

Furthermore, H(ψ) will only be computed for subjects who are still alive at time $t_{k}$ with

(B4)
$$H(\psi ,k)=t_{k}+\int_{t_{k}}^{Y}\exp\{\psi A(u)\}du.$$

The mapping from $\left\{Y,{Ā}_{k},{\bar{L}}_{k}\right\}$ to $\left\{H(\psi ,k),{Ā}_{k},{\bar{L}}_{k}\right\}$ is one to one.

Then, the distribution of the treatment regime $A_{k}$ can be modeled parametrically. For example, one can consider a pooled logistic regression for the binary treatment A:

(B5)
$$\mathrm{logit}(P(A_{k}=1|{\bar{L}}_{k},{Ā}_{k-1}))=\alpha_{0}+\alpha_{1}L_{k}+\alpha_{2}A_{k-1}.$$

The above model appears simple because it relies on the Markov assumption that the treatment probability at visit k is associated only with the most recent treatment/covariate history.

Next, SRA ensures:

(B6)
$$f\{A_{k}|{\bar{L}}_{k},{Ā}_{k-1},H(\psi^{*},k)\}=f\{A_{k}|{\bar{L}}_{k},{Ā}_{k-1}\}.$$

This assumption suggests that, if add $H(\psi^{*},k)$ as a covariate to the treatment distribution parametric model (e.g., add $\theta H(\psi^{*},k)$ to RHS of Formula (B5)), the coefficient of this term (e.g., θ) should be zero. Here, $\psi^{*}$ denotes the true value of the causal parameter ψ As a result, an asymptotic α‐level G‐test of the hypothesis $\psi =\psi^{*}$ is performed by using any standard likelihood‐based test (e.g., a score test) of the hypothesis θ=0 based on the likelihood of the exposure function. The 95% confidence interval of ψ is the set of ψ when the hypothesis that θ=0 cannot be rejected. In conclusion, the G‐estimation searches for the ψ that recovers the latent unexposed counterfactuals, which are conditionally independent of $A_{k}$ given histories, at each k.

Essentially, estimating the causal parameters in the structural models is equivalent to solving the estimating equation

(B7)
$$U(\psi )=\overset{N}{\underset{i=1}{\sum }}U_{i}(\psi ,\hat{\alpha })=0,$$

where ψ is the causal parameter of interest (e.g., the causal survival time ratio) and $\hat{\alpha }$ is the consistent estimator of a p‐dimensional nuisance parameter α which describes the treatment mechanism parametric distribution. For the running example with binary treatment, the explicit G‐estimating equation is:

(B8)
$$U(\psi )=\overset{N}{\underset{i=1}{\sum }}\overset{V_{i}}{\underset{k=0}{\sum }}H_{i}(\psi ,k)\{A_{i,k}-Pr(A_{i,k}=1|{\bar{L}}_{i,k},{Ā}_{i,k-1})\}=0.$$

For high‐dimensional cases such as $\psi =\{\psi_{1},\psi_{2},\cdots \;,\psi_{\nu }\}$, ν estimating equations can be constructed and a ν degree‐of‐freedom $\chi^{2}$ test can be used to test the conditional independence [4, 11].

Using T=min(Y,C) instead of Y when right censoring is present will introduce bias because treatment is associated with censoring when the null hypothesis is false. Artificial censoring is an accepted solution to correct the bias. This method starts with replacing the event time $Y_{i}$ in Equation (B4) with the right censoring time $C_{i}$. Consequently, the *minimal potential follow‐up time* is defined as

(B9)
$$C_{i}^{*}(\psi ,k)=\underset{\forall g\in 𝒢}{\min}\left\{t_{k}+\int_{t_{k}}^{C_{i}}\exp\{\psi A(u)\}du\right\}.$$

Now $C_{i}^{*}(\psi ,k)$ is a fixed function of $\left\{{\bar{L}}_{k},{Ā}_{k-1}\right\}$ but not a function of $A_{k}$. The artificial censored bivariate random variable {time, event indicator}: $\left\{X_{i}(\psi ,k),\Delta_{i}(\psi ,k)\right\}$ can be calculated as before but replacing $C_{i}(\psi ,k)$ with $C_{i}^{*}(\psi ,k)$. The newly defined counterfactuals $X_{i}(\psi ,k)=\min(H_{i}(\psi ,k),C_{i}^{*}(\psi ,k))$ and $\Delta_{i}(\psi ,k)=1\{H_{i}(\psi ,k)<C_{i}^{*}(\psi ,k\}$ would restore the exchangeability when $\psi =\psi^{*}$. Thus, the G‐estimation can proceed after artificially censoring some subjects, though their event times were observed.

## Appendix C Hyperparameter Tuning

Similar to other ML algorithms, the hyperparameters in GE‐SCORE and GE‐MIMIC need to be tuned on a case‐by‐case basis. Some important parameters to be considered are discussed in this section, under the context of the simulations presented in the main text. We start with the network that predicts $Pr(A_{k}|{\bar{Z}}_{k})$. Consistency of this model is the key to the success of both GE‐SCORE and GE‐MIMIC. Yet, this is a standard supervised learning problem. A shallow RNN is sufficient, especially when the targeted output (the exposure sequence $\left\{A_{k}\right\}$) is low‐dimensional. More advanced recurrent units, such as LSTM, can easily replace the simple RNN unit. In later discussion, a one‐layer simple RNN with 20 units for the exposure prediction network with sigmoid activation function for the output layer is sufficient.

Given that the input dimension is modest across all simulation scenarios, our experiments indicate that a one‐layer RNN or LSTM with a single hidden neuron is sufficient to capture the relevant temporal structure. More generally, the appropriate complexity of the counterfactual outcome network should be guided by domain knowledge. If one believes that the true causal survival ratio $\psi^{*}$ depends on only a small subset of covariates in a largely linear manner, adopting an overly complex network may unnecessarily increase estimation variance and lead to unstable outputs. On the other hand, if the underlying relationship is believed to be highly nonlinear or to involve complex interactions among covariates, a more expressive network architecture may be required to adequately capture such structure.

The network processes data in batches; consequently, the correlation measurement used in the loss functions is calculated per batch. Thus, both proposed algorithms require a sufficiently large batch size to ensure the batch sample correlation well represents the whole sample correlation. From our simulations, a batch size around 25% of the training sample functioned well for GE‐SCORE. In GE‐MIMIC, we choose the original training sample size as the batch size. Thus, it takes M batches to process all training samples.

The initialization of weights is another important hyperparameter that can be tuned. The stochastic nature of the optimization allows any single run of the algorithm to generate different predictions. Besides fixing the initialization schema to the Glorot normal initializer [27], we propose to run each algorithm multiple times (e.g., 20 times). Initializing the network with different weights allows the algorithm to start from different places, hence providing a larger likelihood of locating the global optima. This is called Multi‐start [28]. The final prediction is the average of the three best runs, evaluated by validation loss. The optimization problem we are facing is non‐convex with a considerable number of local minima. Reporting the mean of multiple runs improves the robustness because the result no longer relies on a single optimization attempt.

Finally, to incorporate a wide range of possible counterfactual causal ratios, we use 4*sigmoid as the activation function for the counterfactual RNN output layer, which allows a range of [0,4] for $\exp(\hat{\psi })$. NAdam optimizer [29] with a learning rate of 0.005 is used for all models. For a real problem that suffers from the curse of dimensionality, tuning becomes more critical.

## Appendix D Simulation Details

The following steps were implemented for individual i to generate a (N=3000) sample.

• *Step 1*: Simulate one baseline static characteristic—gender, which is a binary variable with 1 denoting female, such that Gender ∼Bernoulli(0.5). Simulate baseline survival time $Y^{\bar{0}}∼\text{Weibull}(a,b)$, where the p.d.f of Weibull is defined as $f(x;a,b)=\left(\frac{a}{b}\right){\left(\frac{x}{b}\right)}^{a-1}\exp\left(-{\left(\frac{x}{b}\right)}^{a}\right)$, a=1,b=5. Define $L_{-1}=A_{-1}=0$. Then for each visit k,
  - When k=0, simulate $L_{0}∼N(0,0.5)$. Simulate binary $A_{0}$ by a logistic regression $\mathrm{logit}[Pr(A_{0}=1|L_{0};\beta )]=\beta_{0}+\beta_{1}L_{0}+\epsilon$.
  - When k>0, simulate $L_{k}∼N(\alpha_{1}A_{k-1}+\alpha_{2}L_{k-1}+\alpha_{3}I\{Y^{\bar{0}}<20\},\epsilon )$, where ϵ∼N(0,0.01) $\left\{\alpha_{1},\alpha_{2},\alpha_{3}\}=\{1,0.5,1\right\}$; simulate $A_{k}$ by $\mathrm{logit}[Pr(A_{k}=1|L_{k},A_{k-1};\beta )]=\beta_{0}+\beta_{1}L_{k}+\beta_{2}A_{k-1}+\epsilon$.
  - For the non‐logistic exposure model, replace the logit model with a random sampling from a nonlinear function set.
• *Step 2*: Define the blip function and corresponding $\psi^{*}$ values. For example, $Y^{{ā}_{k-1},\bar{0}}=Y^{{ā}_{k},\bar{0}}\cdot \exp(\psi^{*}A_{k})$. here the true ψ is a constant, unrelated to other covariates.
• *Step 3*: Simulate the event indicator $E_{k}$ and the observed survival time T based on the blip function defined in Step 2, $Y^{\bar{0}}$ and exposure/covariates simulated in Step 1. For example, with a constant $\psi^{*}$, at each k,k=(0,⋯,K):
  if $Y^{\bar{0}}>\int_{0}^{t_{k+1}}\exp(\psi^{*}A(u))du$ then $E_{k}=0$, $T=t_{k+1}$;
  else if $Y^{\bar{0}}\leq \int_{0}^{t_{k+1}}\exp(\psi^{*}A(u))du$ then $E_{k}=1$, $T=t_{k}+[Y^{\bar{0}}-\int_{0}^{t_{k}}\exp(\psi^{*}A(u))du]\cdot \exp(-\psi^{*}A_{k})$.
• *Step 4*: Redefine $L_{k}=0,A_{k}=0$ for all k when $t_{k}>T$. The resultant long‐format data has $V_{i}+1$ valid records for each subject.

In the above simulation, ϵ∼N(0,0.01) is the random noise. Various blip functions hypothesized in Step 2 reflected distinct causal effects on the time to first event. Furthermore, under each scenario, we considered both beneficial/adverse exposure effects. Additionally, the exposure mechanism could be logistic or non‐logistic to test the robustness of methods under exposure model misspecifications.

For G‐estimation, we employed a one‐dimensional ψ estimation which searches on [−1.5,1.5] with a grid size of 0.02; or a two‐dimensional $\left\{\psi_{1},\psi_{2}\right\}$ estimation that searches on ${\left[-1,1\right]}^{2}$ with a step size of 0.05. The exposure mechanism used in the G‐estimation is a logistic regression: $f(A_{k})=\alpha_{k}+\alpha_{1}L_{k}+\alpha_{2}A_{k-1}$. The stabilized weights used in the marginal structural Weibull model are estimated by the R package ipw [30]. Last, GE‐SCORE and GE‐MIMIC simply take $\left\{L_{k},A_{k-1},Gender\right\}$ as input, for both counterfactual and exposure networks under all scenarios.

Table D1 presents the mean and standard deviations of the absolute estimation bias of 100 repetitions, under four scenarios defined in Section 4.1.

**TABLE D1:** Simulation results.

Causal effect/ Exposure mechanism	Estimation method	Scenario I^a^ absolute bias mean (s.d.)	Scenario II^b^absolute bias mean (s.d.)	Scenario III^c^absolute bias mean (s.d.)	Scenario IV absolute bias mean (s.d.)
Beneficial/Logistic	G‐est 1‐*d*	0.049 (0.033)	0.373 (0.028)	0.365 (0.029)	0.233 (0.026)
	G‐est 2‐*d* (L)	0.111 (0.073)	0.084 (0.058)	0.176 (0.055)	0.297 (0.064)
	G‐est 2‐*d* (Gender)	0.020 (0.032)	0.302 (0.021)	0.285 (0.019)	0.197 (0.025)
	Weibull MSM	0.053 (0.039)	0.287 (0.008)	0.298 (0.011)	0.184 (0.017)
	GE‐SCORE (simple RNN)	0.078 (0.055)	0.230 (0.041)	0.263 (0.038)	0.168 (0.033)
	GE‐SCORE (LSTM)	0.133 (0.080)	0.217 (0.045)	0.252 (0.038)	0.157 (0.035)
	GE‐MIMIC (simple RNN)	0.071 (0.042)	0.203 (0.040)	0.243 (0.035)	0.159 (0.036)
	GE‐MIMIC (LSTM)	0.129 (0.029)	0.236 (0.038)	0.237 (0.033)	0.148 (0.034)
Beneficial/Non‐Logistic	G‐est 1‐*d*	0.052 (0.048)	0.327 (0.053)	0.345 (0.049)	0.219 (0.037)
	G‐est 2‐*d* (L)	0.196 (0.187)	0.224 (0.288)	0.305 (0.267)	0.362 (0.139)
	G‐est 2‐*d* (Gender)	0.029 (0.039)	0.285 (0.035)	0.269 (0.041)	0.193 (0.037)
	Weibull MSM	0.062 (0.048)	0.264 (0.024)	0.276 (0.025)	0.171 (0.023)
	GE‐SCORE (simple RNN)	0.076 (0.051)	0.233 (0.058)	0.258 (0.048)	0.155 (0.037)
	GE‐SCORE (LSTM)	0.091 (0.065)	0.219 (0.047)	0.250 (0.051)	0.153 (0.044)
	GE‐MIMIC (simple RNN)	0.087 (0.067)	0.224 (0.057)	0.251 (0.041)	0.168 (0.050)
	GE‐MIMIC (LSTM)	0.075 (0.049)	0.216 (0.053)	0.243 (0.044)	0.152 (0.037)
Harmful Logistic	G‐est 1‐*d*	0.057 (0.046)	0.295 (0.017)	0.286 (0.017)	0.156 (0.021)
	G‐est 2‐*d* (L)	0.105 (0.078)	0.138 (0.074)	0.182 (0.039)	0.265 (0.026)
	G‐est 2‐*d* (Gender)	0.080 (0.045)	0.290 (0.017)	0.261 (0.018)	0.161 (0.021)
	Weibull MSM	0.040 (0.031)	0.265 (0.008)	0.262 (0.011)	0.143 (0.016)
	GE‐SCORE (simple RNN)	0.058 (0.036)	0.223 (0.033)	0.233 (0.028)	0.125 (0.023)
	GE‐SCORE (LSTM)	0.059 (0.044)	0.218 (0.034)	0.229 (0.029)	0.127 (0.028)
	GE‐MIMIC (simple RNN)	0.052 (0.039)	0.204 (0.034)	0.216 (0.036)	0.125 (0.023)
	GE‐MIMIC (LSTM)	0.096 (0.027)	0.198 (0.031)	0.213 (0.026)	0.120 (0.027)
Harmful Non‐Logistic	G‐est 1‐*d*	0.066 (0.060)	0.288 (0.026)	0.284 (0.019)	0.157 (0.028)
	G‐est 2‐*d* (L)	0.194 (0.198)	0.206 (0.149)	0.214 (0.089)	0.374 (0.081)
	G‐est 2‐*d* (Gender)	0.093 (0.064)	0.277 (0.021)	0.252 (0.023)	0.164 (0.027)
	Weibull MSM	0.045 (0.039)	0.249 (0.016)	0.250 (0.013)	0.142 (0.024)
	GE‐SCORE (simple RNN)	0.068 (0.041)	0.222 (0.044)	0.236 (0.037)	0.131 (0.028)
	GE‐SCORE (LSTM)	0.070 (0.052)	0.226 (0.048)	0.233 (0.042)	0.130 (0.034)
	GE‐MIMIC (simple RNN)	0.064 (0.041)	0.201 (0.046)	0.215 (0.026)	0.122 (0.030)
	GE‐MIMIC (LSTM)	0.067 (0.046)	0.201 (0.047)	0.218 (0.036)	0.118 (0.030)

Abbreviations: s.d, standard deviation; MSM, marginal structure model.

^a^ Scenario I: $\psi_{1}=-0.5$ for beneficial causal effect and $\psi_{1}=-0.5$ for harmful causal effect.

^b^ Scenario II: $\psi_{1}=0,\psi_{2}=-0.3$ for beneficial causal effect and $\psi_{1}=0,\psi_{2}=0.3$ for harmful causal effect.

^c^ Scenario III: $\psi_{1}=0,\psi_{2}=-0.2,\psi_{3}=-0.2,\psi_{4}=-0.1$ for beneficial causal effect and $\psi_{1}=0,\psi_{2}=0.2,\psi_{3}=0.2,\psi_{4}=0.1$ for harmful causal effect.
